# Does Posterior Cruciate Ligament Retention or Sacrifice in Total Knee Replacement Affect Proprioception? A Systematic Review

**DOI:** 10.3390/jcm10163470

**Published:** 2021-08-05

**Authors:** Marco Bravi, Fabio Santacaterina, Federica Bressi, Rocco Papalia, Stefano Campi, Silvia Sterzi, Sandra Miccinilli

**Affiliations:** 1 Department of Physical and Rehabilitation Medicine, Università Campus Bio-Medico di Roma, Via Alvaro del Portillo, 200, 00128 Rome, Italy; f.santacaterina@unicampus.it (F.S.); f.bressi@unicampus.it (F.B.); s.sterzi@unicampus.it (S.S.); s.miccinilli@unicampus.it (S.M.); 2 Department of Orthopaedic and Trauma Surgery, Università Campus Bio-Medico di Roma, Via Alvaro del Portillo, 200, 00128 Rome, Italy; r.papalia@unicampus.it (R.P.); s.campi@unicampus.it (S.C.)

**Keywords:** proprioception, total knee replacement, total knee arthroplasty, posterior cruciate ligament, joint position sense, balance, joint kinesthesia

## Abstract

Background: Proprioception is an important part of the somatosensory system involved in human motion control, which is fundamental for activities of daily living, exercise, and sport-specific gestures. When total knee arthroplasty (TKA) is performed, the posterior cruciate ligament (PCL) can be retained, replaced, or discarded. The PCL seems to be responsible for maintaining the integrity of the joint position sense (JPS) and joint kinesthesia. The aim of this review was to assess the effect of PCL on knee joint proprioception in total knee replacement. Methods: This systematic review was conducted within five electronic databases: PubMed, Scopus, Web of Science, Cochrane, and PEDro with no data limit from inception to May 2021. Results: In total 10 publications were evaluated. The analysis was divided by proprioception assessment method: direct assessment (JPS, kinesthesia) and indirect assessment (balance). Conclusions: The current evidence suggest that the retention of the PCL does not substantially improve the joint proprioception after TKA. Due to the high heterogeneity of the studies in terms of design, proprioception outcomes, evaluation methods, further studies are needed to confirm the conclusions. In addition, future research should focus on the possible correlation between joint proprioception and walking function.

## 1. Introduction

Proprioception is an important part of the somatosensory system that can be defined, in relation to the joints, as “the perception of joint movement as well as the position of the body segments in space” and the “perceptions of relative flexions and extensions of our limbs” [1]. In orthopedic surgery the term proprioception is generally defined as the ability of the individual to perceive the position of a joint in space [2]. A unanimously agreed definition of proprioception has not yet been established although we can summarize it as the body’s own sense of position and motion, which includes body segment static position, displacement, velocity, acceleration, and muscular sense of force [3,4]. Proprioception is a fundamental pillar of human motion control, which is essential for activities of daily living, exercise, and sports specific gesture [5]. Indeed, joint proprioception guarantees reflex responses that protect the joint from excessive and potentially harmful movements, maintenance of joint stability during static positions and coordination of joint movements [6,7].

Various techniques are reported in the literature to evaluate joint proprioception [5,8]. The most relevant are [5]:

- Threshold to detection of passive motion (TTDPM): the subject is asked to indicate when he perceives the joint movement that is passively performed by a mobilization system starting from a stationary position.
- Joint position reproduction (JPR), or active joint position detection (AJPD): the subject is required to actively reproduce an established joint angle. This test allows calculation of the accuracy of the replication of the joint angle.
- Active movement extent discrimination assessment (AMEDA): the subject is asked to perform and recognize predetermined knee flexion positions.

Total knee arthroplasty (TKA) represents the definitive treatment in knee osteoarthritis (OA) [9]. The number of TKAs in the United States is expected to increase 143% by 2050 [10]. Furthermore, a recent study estimated an increase up to 91% and 155%, respectively, for primary and revision TKAs by 2030 in Korea [11]. When TKA is performed, the surgeon can retain or sacrifice structures such as the posterior cruciate ligament (PCL) [12]. One of the goals of the orthopedic surgeon when performing a TKA is to reproduce natural knee movements while maintaining stability in the whole range of movement [13,14]. The PCL, through different types of mechanoreceptors, seems to be responsible for maintaining the integrity of the joint position sense and joint kinesthesia [15,16]. Different proprioceptive receptors (e.g., muscle spindles, Golgi tendon organs, Golgi receptors, Pacinian corpuscles, Ruffini endings and bare nerve endings) are present in the structures of the knee (e.g., muscles, tendons, ligaments, menisci, and capsule), sending afferent signals and are responsible for joint proprioception of the knee [6].

Based on a Cochrane systematic review and meta-analysis [12] investigating the difference between either retention or sacrifice of the PCL on major outcomes such as range of motion, knee pain, implant survival rate, validated clinical and functional questionnaire scores (i.e., Western Ontario and McMaster Universities Osteoarthritis Index (WOMAC), patient satisfaction, complications, re-operations other than revision surgery (e.g., manipulation because of impaired knee function) no clear and relevant differences were identified. However, the effects on proprioception have not been investigated.

More recent systematic reviews and meta-analysis by Di Laura Frattura et al. [7] and by Bragonzoni et al. [17] investigated the effects of TKAs on joint proprioception with conflicting results. In fact, although Di Laura Frattura et al. [7] found that proprioception in OA patients undergoing TKA improves but remains impaired after surgery, Bragonzoni et al. [17] found no consensus in the literature about the improvement or worsening in proprioception before and after TKA. However, these reviews did not focus on the effect of PCL retaining or sacrifice on proprioception.

The purpose of this systematic review is to understand whether the proprioception of subjects who have undergone TKA are influenced by the sacrifice (PS design) or retention (CR design) of the PCL. In the present paper we separately analyzed the studies that directly evaluate knee proprioception (as described above) and those that assessed proprioception indirectly through static and dynamic balance evaluation.

## 2. Materials and Methods

### 2.1. Systematic Literature Search

An online systematic search was performed on 26 May 2021, using the following electronic databases: PubMed/MEDLINE, Scopus, Web of Science (WOS), Cochrane, and PEDro. No data limit was used. The Population Intervention Comparison and Outcome (PICO) model was adopted to conduct an evidence-based practice literature search [18] (Table 1). The search strategy used a combination of medical subject heading (MeSH) terms and free-text terms adjusted according to each database specification. An additional manual search and a reference lists examination was performed. The search strategy is shown in Appendix A. The review protocol has been registered in PROSPERO (registration number: CRD42021259569).

### 2.2. Data Extraction

**Figure 1:**
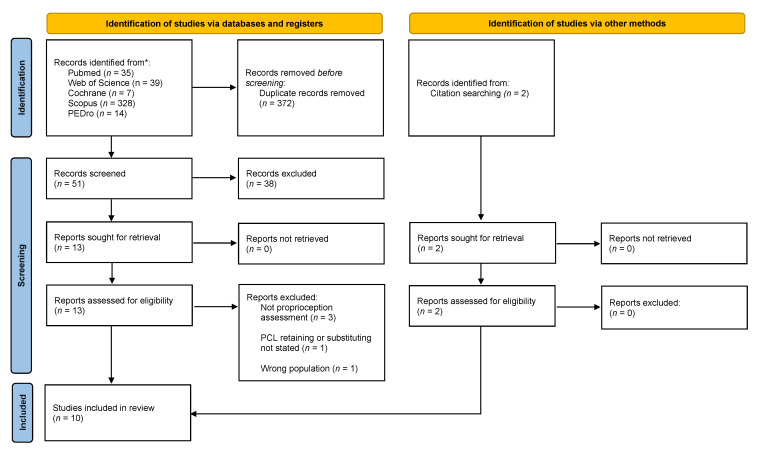
Selection flow diagram according to the PRISMA 2020 statement. From: Page, M.J.; McKenzie, J.E.; Bossuyt, P.M.; Boutron, I.; Hoffmann, T.C.; Mulrow, C.D.; Shamseer, L.; Tetzlaff, J.M.; Akl, E.A.; Brennan, S.E.; et al. The PRISMA 2020 statement: an updated guideline for reporting systematic reviews. *BMJ* **2021**, *372*, n71, doi:10.1136/bmj.n71. For more information, visit: http://www.prisma-statement.org/ (accessed on 13 May 2021).

The Preferred Reporting Items for Systematic Reviews and Meta-analysis (PRISMA) guidelines were used (Figure 1) [19]. Only English written original full-text articles, published from inception to May 2021 about the assessment of proprioception in adult patients with CR or PS TKA were included in this review. Exclusion criteria were non-clinical studies, abstract, editorial, review article, book chapter, case report, not assessed proprioception in the knee joint, focused on partial knee replacement.

Duplicated references were manually identified and excluded, then two reviewers (MB, FS) independently screened title and abstracts to identify eligible articles. At the end of this phase the two reviewers met to discuss the inclusion of all articles that were selected by a single reviewer.

Subsequently, the full texts of the selected articles were screened by both reviewers to verify if they met the inclusion criteria and the presence of any exclusion criteria. After the selection of eligible studies, data were extracted, including the name of the first author, year of publication, study design and objective, characteristics of the participants, (e.g., mean age, male prevalence), the type of equipment used to proprioceptive evaluation, proprioceptive outcome, evaluation protocol and summary of results. Any discrepancies that occurred in any of the phases described were discussed with a third reviewer (SM).

### 2.3. Quality Assessment

The methodological quality of included studies was evaluated by two independent reviewers (MB, FS), a third reviewer (SM) was consulted if discrepancies were not resolved by discussion. The tools for the methodological quality assessment of the included studies were chosen according to Ma et al. [20]. Randomized controlled trials (RCTs) were assessed using the version two of the Cochrane risk-of-bias tool for randomized trials (RoB 2) [21]; non-RCT studies were assessed using the MINORS (Methodological Index for Nonrandomized Studies) checklist [22], a tool specifically developed to assess the quality of nonrandomized surgical studies. It includes 12 items; the last 4 are specific for comparative studies. The score for the items varies from 0 to 2 (0, not reported; 1, reported but poorly reported or inadequate; 2, reported but well reported and adequate). The maximum global score is set to 16 for a noncomparative study and 24 for a comparative study.

Since a meta-analysis could not be performed due to the lack of homogeneity of proprioception assessment, a best evidence synthesis was performed according to van Tulder et al. [23]. The evidence level was then stated through the following rules: strong evidence if 2 or more studies with low risk of bias and generally consistent findings in all studies (≥75% reporting consistent findings) were found; moderate evidence if 1 study with low risk of bias and 2 or more moderate/high risk of bias studies or with 2 or more moderate/high risk of bias and generally consistent findings in all studies (≥75%) were found; limited evidence if 1 or more studies with moderate/high bias risk or 1 low bias risk study and generally consistent findings (≥75%) were found, conflicting evidence with conflicting findings (<75% of the studies reporting consistent findings).

## 3. Results

### 3.1. Systematic Literature Review Synthesis

A total of 423 articles were identified through a systematic review of the literature (Figure 1). After removing 372 duplicates, a total of 51 titles and abstracts studied were examined and 38 studies were excluded because they did not meet our inclusions criteria. A total of 13 studies plus 2 studies retrieved through citation searching were assessed for eligibility. After full-text reading, 10 studies [16,24,25,26,27,28,29,30,31] were included in the qualitative analysis of this systematic review. Non-randomized studies [16,24,26,27,28,29,30,31,32] showed an average moderate risk of bias assessed through the MINOR scale (Table 2), the RCT [25] showed an overall low risk of bias assessed through Rob2.

The study population consisted of 383 patients, including 262 females and 121 males; age ranged from 26 to 91 years. The analysis of the studies showed heterogeneity in the method of assessing proprioception: two studies [24,29] assessed proprioception through an indirect method (e.g., the ability to maintain balance on both or a single leg), six studies [16,26,27,28,30,31] used a direct method of assessing proprioception (e.g., JPS, TTDPM), one study [25] used both direct and indirect methods. Details of the included studies are summarized in Table 3.

### 3.2. Indirect Proprioception Assessment

Four papers [24,25,29,32] analyzed the effects on proprioception of CR versus PS TKA using an indirect assessment technique. Two studies [25,29] used the Biodex Balance System^®^, two studies [24,32] used the Balance Master System^®^. The postural stability tests were performed in different ways: two studies [24,29] analyzed the ability to maintain balance both in a bipodal stance position and on a single leg, and two studies [25,32] used assessed the ability to balance only in the bipodal stance position.

The comparison with the unaffected contralateral leg was evaluated in two studies [24,29] and showed that about 5 months after surgery, regardless of the type of TKA design, proprioception is non significantly different to that of the contralateral (moderate evidence). The single leg test showed no significant differences between the CR and PS group. The balance test on both legs did not show significant differences between CR and PS in any of the studies analyzed (moderate evidence).

Only Swanik et al. [25] compared the effects with respect to preoperative assessment, regardless of the type of intervention reported a significant (*p* < 0.05) improvement in balance (limited evidence).

### 3.3. Direct Proprioception Measurement

Direct evaluation of joint proprioception was performed using JPS and joint kinesthesia assessments through TTDPM. A total of four studies [16,27,28,30] assessed the JPS, two studies [26,31] assessed the TTDPM, and one study [25] assessed both tests.

The JPS was evaluated using different methods: one study [16] evaluated the JPS by asking the subject to reproduce on a hand-held model of the leg the degree of flexion of the knee passively positioned by a machine. Three studies [27,28,30] evaluated the JPS by means of the joint position reproduction (JPR) test, one study [28] performed the test with the patient in standing position, while the other two studies [27,30] carried out the test with the patient in a sitting position with the legs hanging freely; in one study [30] the leg was passively positioned by the examiner at the target position, in two studies [27,28] the patient was asked to actively reach the target position and then reproduce it. Finally, Swanik et al. [25] assessed the JPS by asking the subject to report when he felt that the knee, passively rotated at a constant speed, had reached the previously reported target position. The studies also reported heterogeneity in the tools used to measure JPS; two studies [16,25] used a non-commercial proprioceptive testing device which passively rotated the knee, one study [30] used a motion capture system (Kinemetrix, Orthodata, Lüdenscheid, Germany) and two studies [27,28] used an electrogoniometer.

The comparison with the contralateral non-operated leg was evaluated in two studies [27,30] which showed no significant differences between the two legs in the JPR test (moderate evidence).

Four studies [16,25,27,28] made a direct comparison between the CR and PS group, while one study [30] compared only the type of CR prosthesis with a healthy control group. The analysis of the studies that directly compared the CR and PS groups showed conflicting results, indeed Swanik et al. [25] found that the PS group was significantly more accurate in one JPS test (when moving into extension form 45° of knee joint flexion) than the CR group, Warren et al. [16] on the contrary found a significantly better result in the CR group, while Lattanzio et al. [27] and Ishii et al. [28] found no significant differences between the two groups (conflicting findings).

The ability to detect joint motion through TTDPM was assessed using a non-commercial device which passively rotated the knee. The comparison between the operated leg and the contralateral non-operated leg was assessed in two studies [26,31], which did not show significant differences for both CR and PS group in the ability to detect joint movement at an average follow-up greater than 23 months after surgery (moderate evidence). Analysis of the results between CR and PS groups did not show significant differences in any of the three studies [25,26,31] (moderate evidence).

## 4. Discussion

This paper reviewed published studies that analyzed the effects of prosthetic design (cruciate-retaining or cruciate-scarifying) on proprioception, to provide an overview of how preserving or scarifying the PCL would influence changes in knee proprioception in patients with OA underwent to TKA. Only 10 studies out of 384 fulfilled the inclusion criteria. Among these four papers assessed proprioception through indirect method, while six papers proposed a direct method of assessment.

Indirect methods were mainly based on balance assessment, the balance according to Baumann et al. [33] and Bragonzoni et al. [17] can really be considered as an indirect measure of proprioception, although it is necessary to underline that, as also reported by Gotz et al. [29], the use of only the two-leg stance position is controversial, since the results could be influenced by both the operated and the contralateral knee. Only Swanik et al. [25] performed a pre-post-surgery evaluation with an indirect method, showing no substantial proprioceptive advantages in preserving PCL. Gotz. et al. [29] and Vandekerckhov et al. [24] showed an improvement of proprioception regardless of the type of prosthesis, both in comparison to the healthy contralateral leg and in the bipodalic test, concluding that the PCL does not seem to play a decisive role in balance control.

The results of studies that used direct methods of joint proprioception and kinesthesia assessment are conflicting. However, a specific comparison between the studies was not possible due to the high heterogeneity in terms of study design, evaluation methods, parameters evaluated and length of follow up.

It has been suggested that the sensory denervation of the PCL begins even before surgery in the osteoarthritic knee [25]. Furthermore, the PCL is not the only structure involved in maintaining knee proprioception. The proprioception of the knee is guaranteed by the integrity of multiple anatomical structures (i.e., muscles, tendons, ligaments, capsule) and by the integration of the different afferent signals coming from the different proprioceptive receptors [6] as well as by signals coming from outside the joint itself (e.g., vestibular and visual system). Therefore, the improvements that are observed are more likely due to be related to the reduction in pain, swelling, deformity, and to the reduced physical activity already described as a possible cause for impaired proprioceptive accuracy in the non-symptomatic knees [6,34] rather than a PCL repopulation by mechanoreceptors [25,35,36,37]. Indeed, regardless of the intervention on the PCL, TKA improves joint space, soft tissue tension and by reducing pain and inflammation allowing a return to daily physical activity that could contribute to knee proprioceptive improvements [2,17]. In support of this hypothesis, it should be emphasized that the muscle spindles, located within muscle fibers, seem to be the most important proprioceptive receptors of the knee particularly involved at mid-range of knee angle [34,38], therefore an improvement of the periarticular muscles structures after TKA and rehabilitation could also be responsible for the improvement of joint proprioception regardless of the surgical involvement of the PCL.

Another aspect that deserves further studies is to understand whether prosthetic design plays a role in proprioceptive recovery after TKA. Indeed, when the PCL is sacrificed, several TKAs design are available to compensate for the absence of the PCL. The PS design is most commonly used and provides a cam post mechanism to cover the functions of the PCL, others design use deep dish inserts with a high anterior rim as a brake against posterior subluxation of the tibia [12]. When the PCL is retained, the use of prosthetics designed with high geometric conformity to the medial articular surface has been found to increase the postoperative maximum flexion angle and ROM [39]. These aspects could influence the results in terms of proprioception and could guide the surgeon in choosing the type of surgery, in fact to date there is still no consensus on when to perform a PCL retaining or sacrifice; Lombardi et al. [13] proposed a decision algorithm for the retention or sacrifice of the PCL based on the patient’s clinical history, clinical evaluation and intraoperative findings. However, the decision to perform a PCL retention or sacrifice is still linked to the state of the ligament, the presence of knee deformity, the type of implant used or the surgeon’s personal preference [12]. In fact, to date, there is still no evidence in favor of PCL retaining or sacrifice as also reported by a recent meta-analysis by Migliorini et al. [40].

Some limitations of the studies included in this review need to be addressed: only one study [25] was an RCT with good methodological quality, all the other studies were found to be lacking from a methodological point of view; most of the studies are retrospective observational studies that do not allow a pure comparison between two homogeneous populations nor a pre–post-surgery comparison; the sample size in several studies was found to be small and inadequately calculated prior to the start of the study; most of the studies did not included a healthy control group and finally the studies analyze proprioception with different methodologies not allowing a comparison between them. Another limitation of this systematic review is the inclusion of only English articles which could lead to an exclusion of relevant studies related to this topic.

## 5. Conclusions

This review study aimed to investigate whether TKAs with PCL retaining or sacrificing could influence proprioception in patients with OA. The heterogeneity of the studies in terms of methodological quality, evaluation instrumentation, and outcome measures hampered the execution of a quantitative analysis through a meta-analysis, however a conclusion can be drawn: this systematic review revealed that patients with knee OA undergoing TKA improves their knee proprioception, and this appears to be regardless of the PCL retention or sacrifice.

According to previous systematic reviews [12,17] we strongly suggest further studies in which trials should be set up to assess consistently the outcome parameters, at uniform time intervals, at least one year follow up should be included if balance assessment is used [17], to make easier future meta-analysis. Furthermore, given the current availability of rapid and valid gait analysis systems [41,42,43], the study of a possible correlation between joint proprioceptive deficits and clinical and functional alterations should be encouraged.

## Figures and Tables

**Table 1:** PICO model.

Population	Intervention	Comparison	Outcome
Adults with total knee replacement	PCL retaining PCL substituting		Direct and indirect proprioception assessment

**Table 2:** MINORS score of non-randomized studies.

	Clearly Defined Aim	Consecutive Patients	Prospective Data Collection	Adequate Endpoint	Blind Assessment	Adequate Length of Follow-Up	Loss to Follow-Up Rate <5%	Prospective Calculation of the Study Size	Control Equivalent to Cases	Control Contemporaneous to Cases	Baseline Equivalence of Groups	Adequate Statistical Analysis	Total Score
Götz et al. (2016)	2	0	2	0	0	2	2	0	2	2	2	2	16/24
Vandekerckhove et al. (2014)	2	2	2	1	1	2	2	0	2	2	2	2	20/24
Bascuas et al. (2013)	2	2	2	1	0	2	1	0	2	2	1	2	17/24
Fuchs et al. (1999)	2	1	2	1	0	2	0	0	2	2	0	2	14/24
Lattanzio et al. (1998)	2	1	2	1	1	2	0	0	1	2	2	2	16/24
Ishii et al. (1997)	1	1	2	1	0	2	1	0	1	2	1	2	14/24
Cash et al. (1996)	2	1	2	1	1	2	2	0	0	2	2	2	17/24
Simmons et al. (1996)	2	2	2	1	0	2	1	0	0	2	2	2	16/24
Warren et al. (1993)	1	1	2	1	0	2	1	0	1	2	1	2	14/24

**Table 3:** Details of included studies.

Author (Year)	Design and Aim	Participants	Instruments	Proprioception Assessment	Assessment Protocol	Results
Götz et al. (2016)	Observational To evaluate whether a CR TKA should be preferred over a PS TKA regarding postural stability in one-leg stance and clinical scores.	40 patients with primary OA designated for TKA. CR group: *n* = 20; 4 males; mean age = 64.3 ± 7.3 y; mean BMI = 31.5 ± 5.9 kg/m^2^; PS group: *n* = 20; 5 males; mean age = 70 ± 7.9 y; mean BMI = 33.4 ± 14.1 kg/m^2^; Assignment to group depending on stability needs. Evaluation mean FU = 5.3 months.	Biodex Balance System^®^ (Biodex Inc., Shirley, NY, USA).	Postural stability by means of ability to balance on both legs and on single leg.	The ability to balance on both legs was tested asking the patients to keep their centre of mass (visible as a moving black dot on a screen in front of them) in the centre of the target for 3 × 20 s. One leg stance: the ability to balance on one leg was tested asking the patients to keep the balance standing with one leg on the locked platform for 3 ×10 s starting with the non-operated leg.	One leg stance: CR operated side vs. healty side: median 1.35 vs. 1.50 (*p* = 0.732). PS operated side vs. healty side: 1.55 vs. 1.60 (*p* = 0.125). No significant differences between groups (2.37 CR, 2.89 PS; *p* = 0.198) of postural stability index (variance of change of platform in degrees) for the two-leg stance on the unstable platform. The difference in one-leg stance was not statistically significant between groups (1.65 CR; 2.05 PS; *p* = 0.314). No proof of the superiority of PCL retaining over substituting in terms of postural stability.
Vandekerckhove et al. (2014)	Observational To perform a functional comparison between CR and PS TKA.	45 patients with TKA for end-stage OA. CR group: *n* = 27; male/female ratio = 0.33; mean age = 70.5 ± 6.4 y; PS group: *n* = 18; male/female ratio = 0.55; mean age = 68.0 ± 8.4 y; Assignment to group depending on stability needs. Evaluation mean FU: CR = 2.9 ± 0.8 years; PS = 3.1 ± 0.8 years.	Balance Master system^®^ (Neurocom, Clackamas, OR, USA)	Balance and postural control tests consisting of five subtests: WBS, mCTSIB, UST, LOS, RWS test.	WBS measures the percentage of body weight undergone by each leg consecutively while standing erect, and then squatting in three positions of knee flexion (30°, 60° and 90°). mCTSIB measures the postural sway velocity in bilateral standing position on the firm and unstable surface with eyes open and closed. UST measures the postural sway velocity on a single leg (both the operated and non-operated leg were tested). LOS quantifies the maximum distance participants can lean their bodies in a given direction without losing balance. Patients are asked to reach 8 targets distributed from a central point and displayed on a screen. RWS quantifies the subject’s ability to rhythmically move their COG from the left to the right and forward to backward at 3 different speeds: slow, moderate and fast.	UST: no significance differences between CR and PS. Comparison of both CR and PS TKA to the non-operated contralateral leg revealed no significant differences. CR group showed statistically higher speed than PS for RWS test at slow (*p* = 0.01) and moderate (*p* = 0.03) speeds. The PCL does not seem to play an important role in balance/postural control.
Bascuas et al. (2013)	Observational	44 (12 male; mean age 71.4 ± 7.12 y; BMI 32.65 ± 5.02 kg/m^2^) patients underwent TKA for severe OA. CR group: *n* = 16 PS group: *n* = 28 Evaluation mean FU: 12 months.	Neurocom Balance Master	Balance, mCTSIB	3 posturopgraphy test: The center of gravity movement is measured 3 times in each test, 10 s per trial. The amount of sway is expressed in degrees per sec. The test was done: on a firm surface with open eyes, on a firm surface with closed eyes, on a foam surface with open eyes, and on a foam surface with closed eyes.	No significant difference existed in posturograpy changes when comparing CR vs. PS group.
Swanik et al. (2004)	Randomized Controlled Trial To evaluate and compare the effects of total knee arthroplasty with cruciate-retaining and posterior stabilized prostheses by assessing proprioception, kinesthesia, and balance	20 patients (13 male) underwent TKA for a grade-2 or 3 OA were randomly assigned to CR or PS group. CR group: *n* = 10; mean age = 71.1 ± 6.3 y;PS group: *n* = 10; mean age = 69.4 ± 5 y; Evaluation at T0 = 1.5 months preoperatively and T1 = 7.6 months postoperatively.	Non-commercial proprioception testing device. Biodex Balance System^®^ (Biodex Inc., Shirley, NY, USA).	TTDPM; JPS; balance test	- TTDPM: the proprioception testing device passively rotated the knee into flexion or extension at a velocity of 0.5°/sec. On perceiving motion, the subject pressed a handheld switch, and the degree of rotation was recorded. Test repeated 6 times: 3 times for passive flexion and 3 time for passive extension. - JPS was measured as reproduction of passive positioning: the subject’s knee was rotated away from the starting position to a presented angle the position was held 10 s. Then, from starting position the knee was passively rotated at a constant velocity (0.5°/sec) toward the presented angle. The subject disengaged the device by pressing the handheld switch when the knee position reproduced the presented angle. The difference between the presented angle and the reproduced angle was recorded in degrees. The test was repeated a total of 4 times, with reproduction of passive positioning moving into flexion and extension measured from both 15° and 45° reference positions. - Balance was assessed asking the subject to maintain balance for 20 s standing on the Biodex unstable platform. The test was repeated 3 times at 2 different levels of difficulty.	PS JPS (mean error, 1.0° ± 0.5°) was significantly more accurate (*p* < 0.05) than CR (mean error, 2.2° ± 1.0°) when moving into extension from 45° knee joint angle. No significant differences for all the other tested positions. TTDPM did not identify any significant differences at either of the two test angles (15° and 45°) moving into either flexion or extension. Balance test: no significant differences between PS and CR group. The results show no substantial proprioceptive or kinesthetic advantages of preserving posterior cruciate ligament.
Fuchs et al. (1999)	Controlled Clinical Trial To evaluate the differences in angle reproduction capability after non-constrained posterior cruciate ligament retaining total knee arthroplasty	28 patients underwent non-constrained CR TKA for OA. CR group: *n* = 28; 11 male; mean age = 65.7 y; CTRL group (healthy): *n* = 25; 11 male; mean age 55.7 y. Evaluation mean FU: 63.9 (range, 13–89) months.	Kinemetrix motion analysis system (Orthodata Lüdenscheid, Germany)	JPS	16 measurements (operated/nonoperated leg: 0°, 30°, 60°, 90°, 90°, 60°, 30°, 0°) were made in the sitting position, with the lower leg hanging free. Patients were blindfolded and not blindfolded. The leg was positioned by the examiner, then relaxed, and afterward, the subject was asked to reproduce the original joint position. The test was repeated 3 times before measurements were taken. The starting joint positions were assumed by the subjects before each measurement and checked by the examiner. The patients were examined starting at 90 and 0° of knee flexion to restore 60° and 30° of knee flexion.	With visual control no significant differences between CR (mean error: 6.1 ± 5.9°) and CTRL (mean error (5.6 ± 4.6°) from 0° to 30° (*p* = 0.734) and from 0° to 60° CR = 11 ± 7.5°; CTRL = 7.2 ± 5°; *p* = 0.033). Without visual control significant differences between CR (7.7 ± 5.9°) and CTRL (4.6 ± 4.7°) (*p* = 0.041). No significant differences between the operated and the contralateral leg. Reduced proprioceptive capabilities are present after non-constrained CR TKA in both the operated and the contralateral leg compared with healthy controls.
Lattanzio et al. (1998)	Observational To examine the role of the PCL in knee-joint proprioception after TKA	20 patients underwent TKA for OA. CR group: *n* = 10; 4 male; mean age = 74 (range, 65–83)y; PS group: *n* = 10; 4 male; mean age = 69 (range, 53–82 y); Evaluation mean FU: CR = 10 (range, 6.3–16.8) months; PS = 12 (range, 6.5–16.0)	Penny and Giles electrogoniometer	JPS	The starting position was 90° of knee flexion and the testing range was 10° to 55° of knee extension from the reference position. 10 randomized test angles were evaluated for each experimental trial. Both the operated and non-operated leg was evaluated. The patient was instructed to extend the testing leg to a predetermined angle. When the test angle was reached, the patient was asked to concentrate on the test angle for 3 s. The patient then lowered the testing leg back to the starting angle and remained there for another 3 s. After the 3-s interval the patient was given 5 s to reproduce the test angle and acknowledge when he/she believed had reproduced the designated test angle. Absolute angular error was measured.	The mean error values between the operated and non-operated knee in each patient were not significantly different (*p* > 0.4). No significant differences between the CR and PS group (*p* > 0.6). Preserving the PCL in TKA may not improve knee-joint proprioception and consequently may not improve functional joint performance after total knee arthroplasty.
Ishii et al. (1997)	Controlled Clinical Trial	48 OA patients undergoing a TKA. 4 male; mean age = 70 (range, 54–82) y. The patients were divided into five study groups. Groups 1–4 included patients with a cemented TKA. Group l: CR and patella not resurfaced. Group 2: CR and patella resurfaced. Groups 3: PS and patella not resurfaced. Group 4: PS and patella resurfaced. Group 5: non-cemented CR TKA and patella notresurfaced. Group 6: age-matched non-arthroplasty mild osteoarthritis patients. Evaluation mean FU: 24 (range, 12–48) months.	A 6-degree-of-freedom electrogoniometer or instrumented spatial linkage.	JPS	The subject actively sets both the initial and repeat angles. The tests were conducted on one leg at a time in randomized order, with the tested leg hanging freely and the patient standing upright on the opposite leg. The resulting error terms were based on the averages of 6 trials, 3 made at 30° and 3 made at 70° of flexion.	Groups 1 and 3 did not have significant difference in either reproducibility of index angle (group 1, 5.6° ± 2.4°; group 3, 5.4° ± 1.6°) or reproducibility of change of angles (group l, 4.6° ± 3.1°; group 3, 5.7° ± 3.7°). For groups 2 and 4, no significant differences in either reproducibility of the index angles (group 2, 6.1° ± 3.1°; group 4, 3.7° ± 1.6°) or reproducibility of the change of angles (group 2, 4.2° ± 3.0°; group 4, 5.1° ± 1.9°). No significant difference among any of the 6 groups for any test at *p* = 0.05. Retaining the PCL does not appear to impart the advantage of improved proprioception.
Cash et al. (1996)	Observational To test the hypothesis that retaining the posterior cruciate ligament during total knee arthroplasty helps preserve the threshold of proprioceptive sensation	60 patients undergone unilateral TKA for OA. CR group: *n* = 30; 13 male; mean age = 69 (range, 69–91) y. PS group: *n* = 30; 9 male; mean age = 65 (range, 41–84) y. Evaluation mean FU: CR = 34 (range, 12–87) months; PS = 39 (range, 12–156) months.	Non-commercial device which passively extended either leg at a constant rate of 0.5° per second.	TTDPM	Patients were seated in a relaxed position with both legs hooked up to the apparatus. Patients were given a right and left sided button and told to hit the button on the side that they perceived motion. Patients were blindfolded not informed as to which leg was being tested. Auditory clues were removed by random initiation of motion by a clutch 5 to 30 s after starting the pulley motors. The knee was tested between 45° and 90° flexion. Each lower extremity was tested 3 times in an independent and random fashion. The recorded angular deflection for each lower extremity was averaged to give a mean angular deflection at the threshold for the operative and nonoperative lower extremities. The patients were asked to indicate their perception of motion by pushing the button for the appropriate extremity.	No difference between CR (2.4 ± 1.0°) and PS (2.4 ± 1.5°) angular deflection. The average difference in angular deflection at threshold between operated and non-operated knees was not significant in both group (*p* = 0.05). The decision to retain the posterior cruciate ligament should not be made based on the premise of improved proprioceptive function.
Simmons et al. (1996)	Observational to quantify whether a difference in proprioceptive ability could be detected between CR TKA and PS TKA.	28 patients (10 male; mean age 69 y) undergone unilateral CR or PS TKA for OA. CR group: *n* = 15; PS group: *n* = 13; Evaluation mean FU: 23 (range, 6–47) months.	A non-commercial proprioception testing device which rotated the knee at a constant angular velocity (0.5°/s), and an optical encoder, which measured angular displacement of the knee in degrees.	TTDPM	The TTDPM was tested from starting positions of 15° knee flexion and 45° knee. The testing device moved the knee randomly into flexion or extension at a constant angular velocity from the two starting positions. The subject signified the detection of passive motion by pressing a remote switch. After 2 practice trials, 3 randomized runs of the TTDPM were subsequently recorded with both flexion and extension from the two starting positions.	No significant difference between mean TTDPM for CR (2.24) and PS groups (2.36). No significant differences between the operated and non-operated knee except for testing condition on 15° moving into flexion (2.44° ± 0.34° vs. 2.01° ± 0.27°, *p* = 0.04). Patients with grade 2 OA: no differences between CR and PS group in TTDPM (*p* > 0.04) Patients with grade 3 OA: PS group performed significantly better than CR at all tests. Retaining the PCL in TKA did not result in improved performance in proprioception testing. Attempting to retain the PCL may be counterproductive in the severely degenerative knee.
Warren et al. (1993)	Controlled clinical trial To test if CR TKA confer better proprioception than PS TKA.	9 healthy subjects (6 male; age range = 26–61 y) and 50 patients undergone TKA for OA (13 male; age range = 56–85 y). A total of 118 knees assessed. CR group: *n* = 25	Electrogoniometer (Penny & Giles Blackwood, Blackwood, Gwent) and a non-commercial apparatus consisting in a well-padded leg supporting jig	JPS	The test involved the passive movemente of the leg to a predetermined sequence of ten positions of knee flexion (0° to 60°). The subject indicated his perception of the position of his knee using a hand-held model of a leg, incorporating another electrogoniometer. Measurements of joint position awareness was performed to measures the inaccuracy in estimating the change of one position to another (mean difference Ω).	Knees with CR TKA showed significantly better (*p* = 0.042) joint position appreciation (mean diff 10.3; range, 4.33–23.67) than PS TKA (mean diff 12.27; range, 4.56–30.1)Irrespective of its precise mechanism, the finding of improved joint position awareness may support the PCL retention in TKA

OA = osteoarthritis; TKA = total knee arthroplasty; FU = follow up; CR = posterior cruciate retaining; PS = posterior cruciate substituting; KSS = knee society score; WOMAC = Western Ontario and McMaster Universities Osteoarthritis Index; ROM = range of motion; KOOS = Knee Injury and Osteoarthritis Outcome Score; HSS = Hospital for Special Surgery score; VAS = Visual Analogical Scale; WBS = weight-bearing squats; UST = unilateral stance test; mCTSIB = modified Clinical Test of Sensory Interaction on Balance; LOS = limit of stability test; RWS = rhythmic weight shift test; TTDPM = threshold to detection of passive motion.

## Appendix A

**Table A1:** Literature search (performed 26 May 2021).

Database	Search Terms
PubMed	(“propriocept*”[All Fields] OR “Proprioception”[MeSH Terms]) AND (“total knee arthroplasty”[All Fields] OR “TKA”[All Fields] OR “TKR”[All Fields] OR “total knee replacement*”[All Fields] OR “arthroplasty, replacement, knee”[MeSH Terms]) AND (“PCL”[All Fields] OR “Posterior Cruciate Ligament”[All Fields] OR “Posterior Cruciate Ligament”[MeSH Terms])
Cochrane	(Propriocept*) AND (“total knee arthroplasty” OR TKA OR TKR OR “total knee replacement*”) AND (PCL OR “posterior cruciate ligament”)
Scopus	ALL(propriocept*) AND (“total knee arthroplasty” OR tka OR tkr OR “total knee replacement*”) AND (pcl OR “posterior cruciate ligament”)
WOS	ALL = (Propriocept*) AND ALL = (“total knee arthroplasty” OR TKA OR TKR OR “total knee replacement*”) AND ALL = (PCL OR “posterior cruciate ligament”)
PEDro ^1^	(Propriocept*) AND (“total knee arthroplasty”)(Propriocept*) AND (“total knee replacement”)

^1^ The research on PEDro was conducted separately with the two strings and then the results were merged. The asterisk (*) is the truncation symbol used at the end of a word to search for all terms that begin with that basic word root.
